# THE MODERATING ROLE OF POSITIVE SOLITUDE IN THE RELATIONS BETWEEN LONELINESS AND DEPRESSIVE SYMPTOMS

**DOI:** 10.1093/geroni/igad104.1058

**Published:** 2023-12-21

**Authors:** Dikla Segel-Karpas, Yuval Palgi, Sharon Ost Mor, Yaira Hamama Raz, Mayan Shacham, Menachem Ben-Ezra, Lee Greenblatt Kimron

**Affiliations:** University of Haifa, Haifa, Hefa, Israel; University of Haifa, Haifa, Hefa, Israel; University of Haifa, Haifa, Hefa, Israel; Ariel University, Ariel, HaMerkaz, Israel; Ariel University, Ariel, HaMerkaz, Israel; Ariel University, Ariel, HaMerkaz, Israel; Ariel University, Ariel, HaMerkaz, Israel

## Abstract

Positive solitude, the choice of being alone to engage in meaningful inner or physical, spiritual, mental, or cognitive activity/ experience, was recently suggested as a stand-alone phenomenon differentiated from loneliness and negative solitude. As loneliness was previously found to have adverse implications for mental health, the present study examined whether the ability to engage in positive solitude can moderate the harmful effect of loneliness on depressive symptoms. The sample consisted of 520 community dwelling older adults in Israel aged 68-87 (Mage=72.66). Participants answered an online questionnaire through a survey company (Ipanel) assessing their background characteristics, depressive symptoms, loneliness, and positive solitude. The results show that loneliness was positively associated with depressive symptoms, whereas positive solitude was negatively associated with depressive symptoms. Furthermore, positive solitude moderated the relationship between loneliness and depressive symptoms, such that higher levels of positive solitude weakened this association. The findings indicate that positive solitude may serve as a buffering factor for mental health among older adults by augmenting coping with the adverse outcomes of loneliness. The results provide insight for tailoring future treatment interventions focusing on positive solitude to enhance mental health among older adults.

